# Opioids prevent regeneration in adult mammals through inhibition of ROS production

**DOI:** 10.1038/s41598-018-29594-1

**Published:** 2018-08-15

**Authors:** Elodie Labit, Lise Rabiller, Christine Rampon, Christophe Guissard, Mireille André, Corinne Barreau, Béatrice Cousin, Audrey Carrière, Mohamad Ala Eddine, Bernard Pipy, Luc Pénicaud, Anne Lorsignol, Sophie Vriz, Cécile Dromard, Louis Casteilla

**Affiliations:** 1 UMR STROMALab, Université de Toulouse, CNRS ERL5311, EFS, INP-ENVT, Inserm U1031, UPS, BP 84225, F-31432 Toulouse Cedex 4, France; 2 Université Paris Diderot, Sorbonne Paris Cité Biology Department, 75205 Paris Cedex 13, France; 3 Centre Interdisciplinaire de Recherche en Biologie (CIRB) CNRS UMR 7241/INSERM U1050/Collège de France 11, Place Marcelin Berthelot, 75231 Paris Cedex 05, France; 4 UMR 152-Pharma-Dev, Université de Toulouse, 31432 Toulouse, France

## Abstract

Inhibition of regeneration and induction of tissue fibrosis are classic outcomes of tissue repair in adult mammals. Here, using a newly developed model of regeneration in adult mammals i.e. regeneration after massive resection of an inguinal fat pad, we demonstrate that both endogenous and exogenous opioids prevent tissue regeneration in adults, by inhibiting the early production of reactive oxygen species (ROS) that generally occurs after lesion and is required for regeneration. These effects can be overcome and regeneration induced by the use of an opioid antagonist. The results obtained in both our new model and the gold standard adult zebrafish demonstrate that this mechanism can be considered as a general paradigm in vertebrates. This work clearly demonstrates that ROS is required for tissue regeneration in adult mammals and shows the deleterious effect of opioids on tissue regeneration through the control of this ROS production. It thus raises questions about opioid-based analgesia in perioperative care.

## Introduction

Tissue fibrosis and regeneration are two opposite forms of tissue repair that take place after injury. While occurring in lower vertebrates and new-born mammals, regeneration after massive resection is largely impaired in adult mammals, which instead exhibit fibrotic healing^1^. As the first line of defence immediately after the injury, inflammation plays a crucial role in the outcome of injury. Inflammation generates a well-known cascade of immune events, among which figures the release of detersive molecules such as reactive oxygen species (ROS) and cytokines^2^. The beneficial effect of ROS on regeneration has been mainly described in the adult zebrafish^3–6^, newt^7^, planarian^8^, gecko^9^ and xenopus tadpole^10^.

After injury, inflammation is also associated with the peripheral release of endogenous opioid peptides by immune cells infiltrating injured tissue and by neural cells^11^. In this context, opioids play both anti-inflammatory and analgesic roles by binding to opioid receptors on immune and neural cells^12,13^. Opioid analogues are therefore commonly used as exogenous agents for systematic peri-operative pain-relief care procedures^14,15^ including inflammatory symptoms and lesions^16–18^. Surprisingly, the consequences of their administration on regeneration have been poorly investigated and conflicting results have been reported in animal models with a moderate epithelium injury^19–22^.

Often investigated and considered as a therapeutic target for its key role in energy homeostasis, white adipose tissue is a complex tissue that displays high plasticity in adults as it can undergo phenotypic (browning) or size (expansion or reduction) modifications depending on the metabolic context^23,24^. It hosts a large pool of regenerative mesenchymal stem/stromal cells that have been widely tested for their regenerative capacities in numerous clinical trials^25,26^. Located just under the skin, subcutaneous inguinal fat pad (IFP) is thus a relevant model for the study of organ plasticity in adult mammals.

We hypothesized that opioids were the key factors directing tissue injury outcome towards regeneration or fibrosis, through their control of ROS production. To test this hypothesis, we developed gain and loss of function experiments in MRL mice, which are well-known for their regenerative capabilities^27^, and in non-regenerative C57BL/6 mice. In a newly developed model of tissue lesion, relying on massive resection of IFP, we show here that, following injury, opioids prevent regeneration by inhibiting ROS production. This mechanism also occurs in the caudal fin of the zebrafish, suggesting that it can be considered as a general paradigm in vertebrates. Altogether, our results provide a new mechanism for the inhibition of regeneration in adults.

## Results

### Massive resection of IFP induces tissue regeneration or fibrosis in MRL and C57BL/6 adult mice respectively

**Figure 1:**
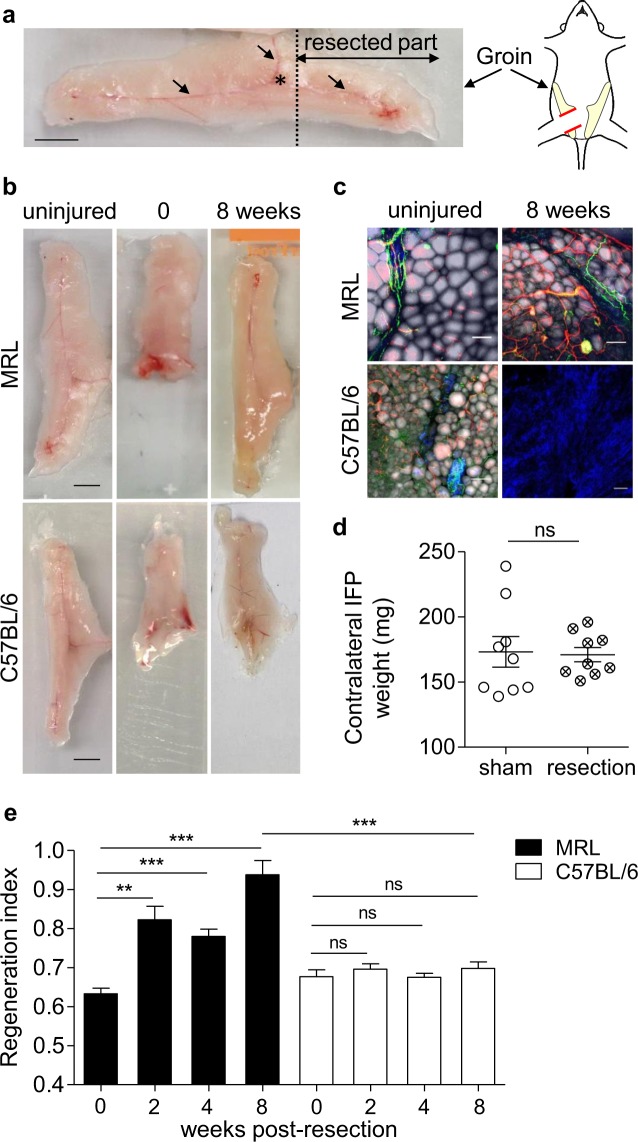
MRL but not C57BL/6 mice can regenerate inguinal fat pad (IFP). (**a**) Macroscopic view of IFP in adult mouse. Dotted line: ablation plane. The lymph node appears as a dark spot (location indicated by a star on the picture) at the crossing of the three main blood vessels (arrows). Scale bar: 0.5 cm. Cartoon; IFP *in situ* localization. (**b**) Macroscopic view of uninjured IFP and IFP at 0 and 8 weeks post-resection in MRL and C57BL/6 mice. Scale bars: 0.5 cm. (**c**) Imaging of uninjured IFP and IFP 8 weeks post-resection, showing adipocytes (BODIPY staining, grey), vascularization (lectin staining, red), sympathetic innervation (tyrosine hydroxylase staining, green) and collagen deposition (second harmonic generation, blue) in MRL and C57BL/6 mice. Scale bars: 100 µm. (**d**) Contralateral IFP weight from sham (○) versus resected C57BL/6 mice (⊗) 8 weeks post-resection. (**e**) Quantification of IFP regeneration in MRL (black) and C57BL/6 (white) mice 0, 2, 4 and 8 weeks post-resection, using the weight ratio (regeneration index) between the resected and the uninjured contralateral IFP. n = 7 to 25 animals per group. Data are represented as mean ± SEM. (ns; not significant, **p < 0.005, ***p < 0.0001). IFP: inguinal fat pad.

To investigate both tissue regeneration and fibrotic healing in mammals, we developed a robust and quantifiable model relying on the massive resection (around 35% of the whole tissue) of the inguinal fat pad (IFP) in adult mice. Using the specific anatomy of the IFP, the resection was systematically performed adjacent to the lymph node, which was used as a visual reference allowing the reproducibility of the resection (Fig. 1a). Macroscopic and microscopic observations as well as IFP weight quantification were performed 8 weeks after surgery. As expected, spontaneous macroscopic regeneration was observed in MRL mice (Fig. 1b upper panel) in contrast to C57BL/6 mice, which did not regenerate (Fig. 1b lower panel). Regenerated IFP exhibited adipocytes, blood vessels and nerves organized in a typical shape and structure similar to the ones observed in the contralateral IFP used as an internal control (Fig. 1c upper panel). In contrast, non-regenerated IFP was characterized by the absence of adipocytes and exhibited fibrotic high collagen deposition (Fig. 1c lower panel). Regeneration was then quantified by the regeneration index (RI), i.e. the weight ratio between the resected IFP and the uninjured contralateral IFP, after a previous check that uninjured IFP weight did not change over the same time following unilateral IFP resection (Fig. 1d). Consistently with macroscopic and microscopic observations, RI was significantly higher in regenerative mice than in non-regenerative mice as early as 2 weeks after resection and the difference was enhanced 8 weeks after resection (0.94 ± 0.037 in MRL vs 0.69 ± 0.017 in C57BL/6 mice) (Fig. 1e). No further regeneration was observed in C57BL/6 mice, even one year after resection. According to these results, this newly developed tissue lesion can be used to decipher regeneration and scarring regulatory processes.

### Opioid signalling controls IFP regeneration

**Figure 2:**
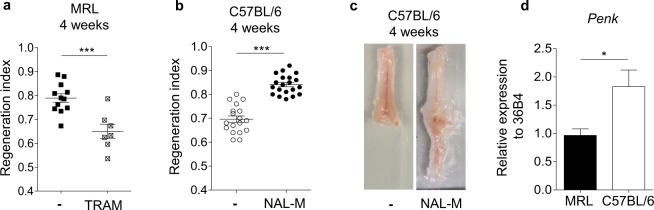
Opioid signalling controls tissue regeneration. (**a**) Quantification of IFP regeneration 4 weeks post resection in MRL mice treated (⊠) or not (■) with TRAM. (**b**) Quantification of IFP regeneration 4 weeks post resection in C57BL/6 mice treated (●) or not (○) with NAL-M. (**c**) Representative images of C57BL/6 mice IFP 4 weeks post resection and NAL-M treatment. (**d**) PENK mRNA expression in MRL (black) and C57BL/6 (white) mice IFP. Data are represented as mean ± SEM. (*p < 0.05, ***p < 0.0005 in a, ***p < 0.0001 in b). NAL-M: naloxone methiodide, TRAM: Tramadol.

To investigate whether opioids inhibited regeneration and/or induced fibrosis, spontaneously regenerative MRL mice and non-regenerative C57BL/6 mice were treated, just after IFP resection, with an opioid receptor agonist (Tramadol, TRAM) or antagonist (naloxone methiodide, NAL-M), respectively. TRAM treatment induced a significant decrease in RI 4 weeks after resection (Fig. 2a; 0.79 ± 0.02 without TRAM vs 0.65 ± 0.030 with TRAM). In contrast, RI was significantly higher in NAL-M treated mice than in untreated mice 4 weeks after resection (RI of 0.84 ± 0.009 with NAL-M vs 0.69 ± 0.013 without NAL-M) (Fig. 2b,c). No change in food intake or body weight was observed, so an indirect effect on feeding behaviour and fat intake regulation could be excluded (Supplementary Figure S1). Altogether, these results demonstrate that opioids inhibit spontaneous tissue regeneration and favour fibrotic tissue formation.

We thus postulated that the absence of spontaneous regeneration in C57BL/6 compared to MRL mice could be associated with a higher synthesis of endogenous opioids in the IFP of C57BL/6 mice. Consistently with this hypothesis, the IFP-expression of proenkephalin (PENK, enkephalin poly-peptide precursor) was significantly higher in IFP of C56BL/6 than in MRL mice (Fig. 2d). In contrast, neither prodynorphin nor pro-opio-melanocortin mRNA was detected in IFP in either mouse strain. These results suggest that inhibition of regeneration in adult mammals could be mediated by endogenous PENK.

### Opioid signalling prevents regeneration through control of ROS levels in zebrafish

**Figure 3:**
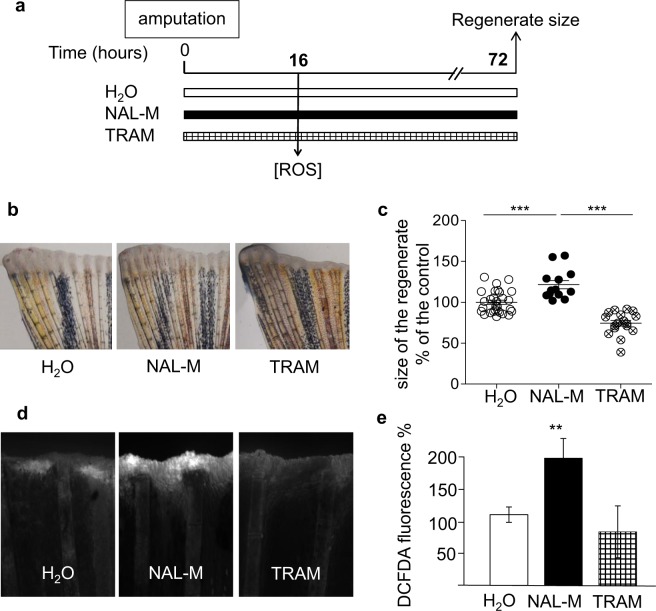
Opioid signalling prevents regeneration through control of ROS production in zebrafish. (**a)** Scheme of the experiment. Caudal fins of adult fish were amputated and then allowed to regenerate for 16 or 72 hours. (**b**) Quantification of the size of the regenerated tissue at 72 hpa (hours post-amputation) in the control (H_2_O) (○) and in fish treated with NAL-M (●) or TRAM (⦻). (**c**) Representative images 72 hpa of caudal fins challenged to regenerate in the presence of NAL-M or TRAM. (**d**) ROS detection (representative images) at the level of the amputation plane at 16 hpa. (**e**) ROS quantification in the control (H_2_O) (white) and in fish treated with NAL-M (black) or TRAM (white checkered). Data are represented as mean ± SEM. (**p < 0.01; ***p < 0.0001). NAL-M: naloxone methiodide, TRAM: Tramadol.

To demonstrate that the anti-regenerative effects of opioids were not tissue and/or model dependent, we used the gold standard adult zebrafish caudal fin regeneration model^28^. Fish were incubated in NAL-M or TRAM from the time of amputation to analysis, and the size of the regenerate was quantified after amputation (Fig. 3a). As in our mouse model, NAL-M enhanced the regenerate size (Fig. 3b,c, 137.7 ± 26%), while TRAM inhibited regeneration (Fig. 3b,c, 70 ± 13%). These results demonstrate that the inhibitory effect of opioids on tissue regeneration is a prevailing process in adult vertebrates.

In zebrafish, regeneration has been widely demonstrated to be controlled by ROS^4^. We thus postulated that opioids controlled caudal fin regeneration through regulation of ROS production. NAL-M treatment enhanced ROS levels at the tip of the amputated fin, the major site of ROS production after amputation (Fig. 3d,e), while TRAM reduced the overall ROS production, even when the area of ROS detection was extended compared to the control (Fig. 3d,e). These results obtained in the caudal fin of zebrafish demonstrate that opioids inhibit tissue regeneration by abolishing the transient peak of ROS.

### Opioid signalling prevents regeneration through control of ROS levels in adult mammals

**Figure 4:**
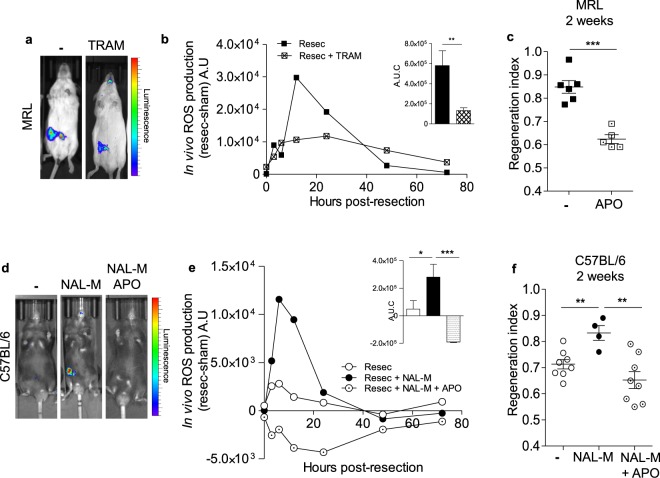
ROS production is required for IFP regeneration. (**a**) Representative *in vivo* imaging of ROS production at 6 hours after surgery in resected MRL mice treated or not with TRAM. (**b**) *In vivo* quantification of ROS production at 0, 3, 6, 12, 24, 48 and 72 hours post-resection in MRL mice treated (⊠) or not (■) with TRAM. n = 8 per group. A.U: arbitrary units. A.U.C: Quantification of ROS production *in vivo* from 0 to 72 hours post-resection in MRL mice treated (white checkered) or not (black) with TRAM. (**c**) Quantification of IFP regeneration in MRL mice 2 weeks post-resection without (■) or with (⊡) APO treatment. (**d**) Representative *in vivo* imaging of ROS production at 6 hours after surgery in resected C57BL/6 mice treated or not with NAL-M or NAL-M and APO. (**e**) *In vivo* quantification of ROS production at 0, 3, 6, 12, 24, 48 and 72 hours post-resection in C57BL/6 mice treated (●) or not (○) with NAL-M or with NAL-M and APO (⨀). n = 8 per group. A.U: arbitrary units. A.U.C: Quantification of ROS production *in vivo* from 0 to 72 hours post-resection in C57BL/6 mice treated (black) or not (white) with NAL-M or with NAL-M and APO (dotted).(**f**) Quantification of IFP regeneration 2 weeks post-resection in C57BL/6 mice treated (●) or not (○) with NAL-M or with NAL-M and APO (⨀). Data are represented as mean ± SEM. (*p < 0.05, **p < 0.005, ***p < 0.0005). APO: apocynin, NAL-M: naloxone methiodide, TRAM: Tramadol.

We therefore investigated whether regeneration in adult mammals was associated with ROS production. In line with this hypothesis, IFP resection induced a robust and transient peak of ROS in the injured tissue of regenerative MRL mice (Fig. 4a,b). ROS production in MRL mice reached maximum values at 12 hours after resection and returned to normal values 72 hours following surgery (Fig. 4b). The treatment of MRL mice with apocynin (APO, inhibitor of NADPH p47 phox subunit translocation^29^) after IFP resection induced a severe decrease in RI two weeks after surgery (Fig. 4c; 0.85 ± 0.027 in untreated mice vs 0.62 ± 0.019 in APO treated mice). These results demonstrate that a robust and transient peak of ROS is required for proper tissue regeneration in adult MRL mice.

The effects of opioids on ROS production were then investigated *in vivo* by treatments with antagonists or agonists of opioid receptors. *In vivo* imaging revealed that the ROS peak observed after resection in IFP of MRL mice was strongly inhibited within 96 hours after TRAM treatment (Fig. 4a,b). In contrast, whereas no peak of ROS was observed in control C57BL/6 mice, NAL-M treatment induced a significant increase in ROS production (Fig. 4d,e). This NAL-M effect on ROS production was abolished after APO treatment (Fig. 4d,e), leading to abolition of a NAL-M effect on RI two weeks post-resection (Fig. 4f). Similar results were obtained using the antioxidant alpha-tocopherol (Supplementary Figure S2). *In vitro* experiments revealed that ROS production under NAL M treatment was specifically increased in immune cells (CD45^+^) and not in non-immune (CD45^−^) cells isolated from IFP stromal vascular fraction (Supplementary Figure S3).

Taken together, these data demonstrate that ROS production is required for proper tissue regeneration in adult mammals and that opioids inhibit this regeneration through the control of ROS production.

## Discussion

By using convergent, complementary models, we have shown, for the first time in adult mammals, that opioids prevent regeneration processes in the early steps post-injury, via the inhibition of an early burst of ROS.

Transient but early use of an opioid receptor agonist is sufficient to inhibit spontaneous regeneration of the IFP in the regenerative, MRL, strain of mice, while treatment with an antagonist is able to induce regeneration in the non-regenerative strain of C57BL/6 mice. This is in line with a very recent study suggesting that administration of the opioid receptor agonist morphine delays pancreatic epithelial regeneration in acute pancreatitis in mice^30^. However, our data also demonstrate that endogenous opioids are key determinants in the control of regeneration. Endogenous PENK expression is lower in regenerative, MRL, than in non-regenerative, C57BL/6, mice. In addition, blocking endogenous opioid receptors (without any administration of exogenous opioids) is sufficient to induce tissue regeneration. The use of naloxone methiodide, which does not cross the blood-brain barrier, and the lack of any effect of opioid treatment on feeding behaviour allow us to exclude involvement of opioid receptors located within the central nervous system and capable of affecting fat pad regeneration through control of food intake and/or energy balance.

We show here that a strong production of ROS by NADPH oxidase occurs both in the spontaneous and the pharmacologically-induced regeneration processes and that its inhibition systematically prevents tissue regeneration in adult mice. We show that this strong production of ROS is a very early and transient phenomenon. The observation that the NADPH oxidase inhibitor and a lipophilic ROS scavenger give similar results allows us to exclude off-target effects of these molecules and reinforces the conclusion that ROS production plays a key role in the outcome of tissue injury. This is the first demonstration of the crucial role of ROS in the control of regeneration in adult mammals. Consistently with our data, Simkin *et al*. observed a stronger production of ROS in the regenerative spiny mouse Acomys than in Mus musculus but without demonstrating their requirement for regeneration^31^. In addition, several reports have highlighted the importance of ROS in regeneration in invertebrates and lower vertebrates^4,10,32^. Altogether, this shows that requiring ROS for proper regeneration is a general paradigm in vertebrates including adult mammals.

Our findings support an anti-oxidative effect (inhibition of ROS production) of endogenous and exogenous opioids *in vivo* that prevents regeneration after tissue lesion. Although we lack a genetic method, the inhibitory role of opioids on regeneration, through the inhibition of ROS production is demonstrated here in different tissues (IFP and caudal fin) and animal models (mouse and zebrafish), showing that this mechanism is neither tissue nor model specific. *In vivo*, such a role has never been described for opioids. An opposite effect of long-term opioid treatment has, however, been described in cultured cells (for review see^33^) and may be explained by the dose and the time of treatment, which could induce desensitization of receptors.

As it is well established that inflammation plays a crucial role in the outcome of injury^34^ and that opioids can affect inflammation^22^, it can be speculated that inflammatory cells may be the link between opioids, ROS production and regenerative processes. Our *in vitro* results indicating that ROS are produced specifically by CD45 positive cells under opioid agonist treatment are consistent with this hypothesis. Inflammatory cells may be the target of opioids and play a key role in the control of regeneration via the production of ROS. Further experiments should be conducted to confirm this hypothesis and clearly identify the cell population subtypes involved.

In conclusion, we have demonstrated a pivotal role of endogenous and exogenous opioids in the tissue repair outcome after lesion in adult vertebrates through the very early inhibition of ROS generation required for regeneration. These effects can be reversed using opioid receptor antagonists, inducing regeneration. Considering that endogenous opioids are potentially released in all peripheral tissues after lesion and that exogenous opioids are largely used in peri-operative care in humans, our results could have broad implications for mammalian tissue repair and regeneration. They thus open up new perspectives for the development of new and pro-regenerative perioperative care protocols that could enhance tissue regeneration while preventing pain.

## Methods

### Animals

All experiments in mice were performed on 5- to 7-week-old male mice. C57BL/6 mice were obtained from Harlan Laboratories. MRL mice (MRL/MPJ) were obtained from The Jackson Laboratory and bred in the CREFRE-US006 (Centre Regional d’Exploration Fonctionnelle et Ressources Expérimentales). Animals were group- housed (3 or 4 per cage) in a controlled environment (12-hour light/dark cycles at 21 °C) with unrestricted access to water and a standard chow diet in a pathogen-free animal facility (IFR150). The animals were maintained in accordance with the guidelines of the European Community Council. Mice were killed by cervical dislocation. All experiments were carried out in compliance with European Community Guidelines (2010/63/UE) and approved by the French ethics committee (protocol reference: CEEA-122 2014-66). Food consumption was measured by food weighing.

Zebrafish colonies (AB-Tu) were maintained using standard methods. The animal facility obtained approval from the French Ministère de l’agriculture (n° C75-05-12), and the protocols were approved by the Ministère de l’education nationale de l’enseignement superieur et de la recherche (00477.02). To maintain a healthy colony, a cycle of 14 h light-10 h dark was used, and a water temperature of 28 °C was maintained, with a maximum density of five fish per litre. Water filtration depended on Aquatic Habitat stand-alone fish housing and operated automatically (Aquatic Habitat, Inc., FL, USA). Fish were fed twice per day with live 2-day-old artemia. For manipulation and amputation, the adult zebrafish (5–10 months of age) were anaesthetized in 0.1% Tricaine (ethyl-m-aminobenzoate), the caudal fins were amputated at the level of the first ray bifurcation and the fins were allowed to regenerate for various lengths of time. The efficiency of regeneration was quantified at 72 hpa (hours post amputation). The surface of the blastema was measured and then divided by the square of the length of the amputation plane for each fish. The efficiency of regeneration was expressed as a percentage of the control.

### IFP resection

Control mice were used for the baseline control and did not undergo surgery. Mice underwent unilateral resection of subcutaneous IFP. Animals were anaesthetized by inhalation of isoflurane 2,5%. After the mice had been shaved, a single incision was made on the abdomen to access and excise 35 to 40% of the right inguinal fat pad between lymph node and groin. The left IFP did not undergo a surgical procedure and was thus used as an internal control. Sham animals were shaved and opened. For these two groups (sham and resected), the skin was closed with 3 suture points. To quantify IFP regeneration, the weight ratio between right (i.e. with resection) and left (i.e. contralateral) fat pads was calculated (regeneration index, RI).

### Immunohistochemistry

IFP sections 300 µm thick were incubated in blocking solution (2% Normal Horse Serum and 0.2% triton X-100 in PBS) at room temperature (RT) and incubated for 24 hours at RT with BODIPY 558/568 (1:1000, D3835, Invitrogen, Carlsbad, CA, USA), tyrosine hydroxylase 488 (1:250, AB1542, Milipore, Darmstadt, Germany) and biotinylated lectin (1:100, B-1105, AbCys, Courtaboeuf, France). Then sections were incubated overnight at 4 °C with streptavidin Alexa-647 (1:100, S21374, Invitrogen, Carlsbad, CA, USA), mounted on a coverslip and imaged using a confocal laser scanning microscope (LSM780, Carl Zeiss, Oberkochen, Germany). Second harmonic generation signal allowing visualization of fibrous collagen was acquired using a mode-locked 890 nm laser and was measured at 445 nm with a 20 nm bandpass filter. Images were processed using Fiji software (NIH, Bethesda, MD, USA).

### *In vivo* treatments

Mice were treated with naloxone methiodide (NAL-M) (subcutaneous injection, 17 mg/kg, N129, Sigma Aldrich, Saint Louis, MO, USA) on days 0–3 after IFP resection. Acetovanillone apocynin (APO) (subcutaneous injection, 100 mg/kg, 200 µl, W508454, Sigma Aldrich, Saint Louis, MO, USA) or alpha-tocopherol acetate (αTOCO) (intraperitoneal injection, 100 mg/mouse, 40 µL, NEPALM) were injected immediately after resection. Tramadol (TRAM) was added to the drinking water (10 mg/kg, Grunenthal, Belgium). Fish were incubated in naloxone methiodide (NAL-M) (5 μM, N129, Sigma Aldrich, Saint Louis, MO, USA) or Tramadol (TRAM) (1 mM, Grunenthal, Belgium).

### *In vivo* ROS imaging

Mice were briefly anaesthetized by inhalation of isoflurane and i.p. injected with 5 mg of luminol (5-amnio-2,3-dihydro-1,4-phtalazinedione, A4685, Sigma Aldrich, Saint Louis, MO, USA) in 100 µl of PBS. The animals’ bioluminescence was imaged using an IVIS Spectrum 200 (Caliper Life Science, Hopkinton, MA, USA) for 2 min exposures at different times after luminol injection. Image analyses were performed using LivingImage 3.0 Software (Caliper Life Science, Hopkinton, MA, USA). The images were colour intensity calibrated from 30 (min) to 330 (max). For each animal, the sham surgery area signal was subtracted from the resected area photon flux.

In zebrafish, 2′,7′-dichlorodihydrofluorescein diacetate (H2DCFDA, Calbiochem, San Diego, CA, USA) was used to monitor the accumulation of ROS in adult zebrafish fins. Fluorescent DCF was formed through ROS oxidation. Zebrafish were incubated with H2DCFDA (50 µM) 2 h prior to confocal imaging. Spinning-disk images were acquired using a 4×/0.15 N.A. objective on a Nikon Eclipse Ti microscope equipped with a CoolSnap HQ2/CCD camera (Princeton Instruments, Trenton, NJ, USA) and a CSUX1-A1 (Yokogawa) confocal scanner. MetaMorph software (Molecular Devices, Sunnyvale, CA, USA) was used to collect the data. Fluorescence was excited with a 491 nm laser and detected with a 525/39 nm filter. Fluorescence intensity was quantified using ImageJ software.

### Cell sorting and *in vitro* ROS quantification

Stromal vascular fraction was prepared from IFP as described by Planat *et al*.^34^ and cells were incubated with 10 µL of anti-CD45 microbeads (Miltenyi Biotec) before magnetic sorting using MACSQuant Tyto (Miltenyi Biotec). CD45^+^ and CD45^−^ cells were maintained in HBSS for ROS quantification.

CD45^−^ and CD45^+^ sorted cell fractions were activated with phorbol 12-myristate 13-acetate (PMA) (100 mM, Sigma Aldrich)) and treated or not with NAL-M (10^−9^ M) for 2 hours before measurement of ROS production by chemiluminescence in the presence of luminol (66 µM, 5-amnio-2,3-dihydro-1,4-phtalazinedione, A4685, Sigma Aldrich, Saint Louis, MO, USA) using a thermostatically monitored luminometer (37 °C) (210410 A EnVision Multilabel Reader). Chemiluminescence was continuously monitored for 1 hour. ROS levels were quantified and expressed using the area under the curve.

### RNA Extraction and Real-Time PCR

For mouse tissues, total RNA was isolated by Qiazol extraction and purified using RNeasy microcolumns (Qiagen). Two hundred and fifty nanograms of total RNA was reverse-transcribed using the High Capacity cDNA Reverse Transcription kit (Life Technologies/Applied Biosystem), SYBR Green PCR Master Mix (Life Technologies/Applied Biosystem), and 300 nmol/L primers on an Applied Biosystem StepOne instrument. PENK relative gene expression was determined using the ∆∆CT method and normalized to *36B4* level.

Primer sequences:

**Table:** 

	Forward primer	Reverse primer
*36B4*	AGTCGGAGGAATCAGATGAGGAT	GGCTGACTTGGTTGCTTTGG
*PENK*	TGCAGCCAGGACTGCGCTAAAT	GATCCTTGCAGGTCTCCCAGAT

### Statistical analyses

Studies were not randomized and investigators were blinded to analyses. All results are given as means ± SEM. Data were analysed using an ANOVA test (when there were more than 2 groups) and variance between groups was compared. Statistical differences were measured using an unpaired two-sided Student’s-t-test, or a nonparametric test (Mann-Whitney) when variances between groups were different. All statistical analyses were performed in GraphPad Prism 5.0 software and a two-tailed P value with 95% confidence interval was acquired.

### Data availability

The datasets generated and/or analysed during the current study are available from the corresponding author on reasonable request.

## Electronic supplementary material

Supplementary Information
