# Supplementary material for: Opioids prevent regeneration in adult mammals through inhibition of ROS production
Source: Sci Rep. 2018 Aug 15;8:12170. doi: 10.1038/s41598-018-29594-1 (PMC6093857; doi:10.1038/s41598-018-29594-1)

Opioids prevent regeneration in adult mammals through inhibition of ROS production

Elodie Labit[1], Lise Rabiller [1], Christine Rampon[2,3], Christophe Guissard[1], Mireille André[1], Corinne Barreau[1], Béatrice Cousin[1], Audrey Carrière[1], Mohamad Ala Eddine[4], Bernard Pipy[4], Luc Pénicaud[1], Anne Lorsignol[1], Sophie Vriz[2,3,*], Cécile Dromard[1,*], Louis Casteilla[1,*]

Supplementary Information

Figure S1: Food intake and body weight are not affected by opioid antagonist treatment.
Figure S2: α-Tocopherol inhibits NAL-M induced ROS production and fat pad regeneration.
Figure S3: Opioid antagonist increases ROS production specifically in CD45 positive cells.

Figure S1: Food intake and body weight are not affected by opioid antagonist treatment. **(a)** Daily food consumption was measured three days before and three days after fat pad resection in mice treated (●) or not (○) with NAL-M for three days following resection. (n=4 animals per group). **(b)** Body weight was measured one month after resection in mice treated (●) or not (○) with NAL-M and in sham mice of the same age treated (▲) or not (Δ) with NAL-M. There is no statistical difference between groups. Data are represented as mean ± SEM. IFP: Inguinal Fat Pad. NAL-M: naloxone methiodide. Resec: fat pad resection.

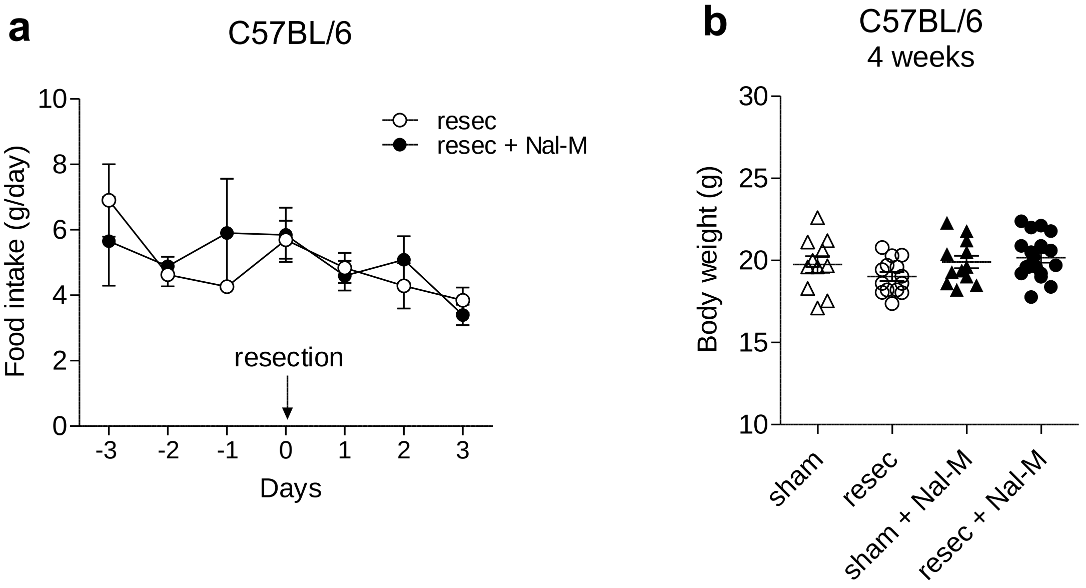

Figure S2: α-Tocopherol inhibits NAL-M induced ROS production and fat pad regeneration.

(a) In vivo quantification of ROS production at 0, 6, 12, 24, 48 and 72 hours post-resection in C57BL/6 mice, untreated (○), treated with NAL-M (●) or treated with NAL-M and αTOCO (◐). A.U.: arbitrary units. A.U.C (area under the curve): Quantification of ROS production in vivo from 0 to 72 hours post-resection in C57BL/6 mice untreated (white bar), treated with NAL-M (black bar) or treated with NAL-M and αTOCO (striped bar). (n=5 animals per group). **(b)** Quantification of IFP regeneration 2 weeks post-resection in C57BL/6 mice, untreated (○), treated with NAL-M (●) or treated with NAL-M and αTOCO (◐). Data are represented as mean ± SEM (* p<0.05, *** p<0.0001). IFP: inguinal fat pad. NAL-M: naloxone methiodide. αTOCO: alpha-tocopherol acetate.

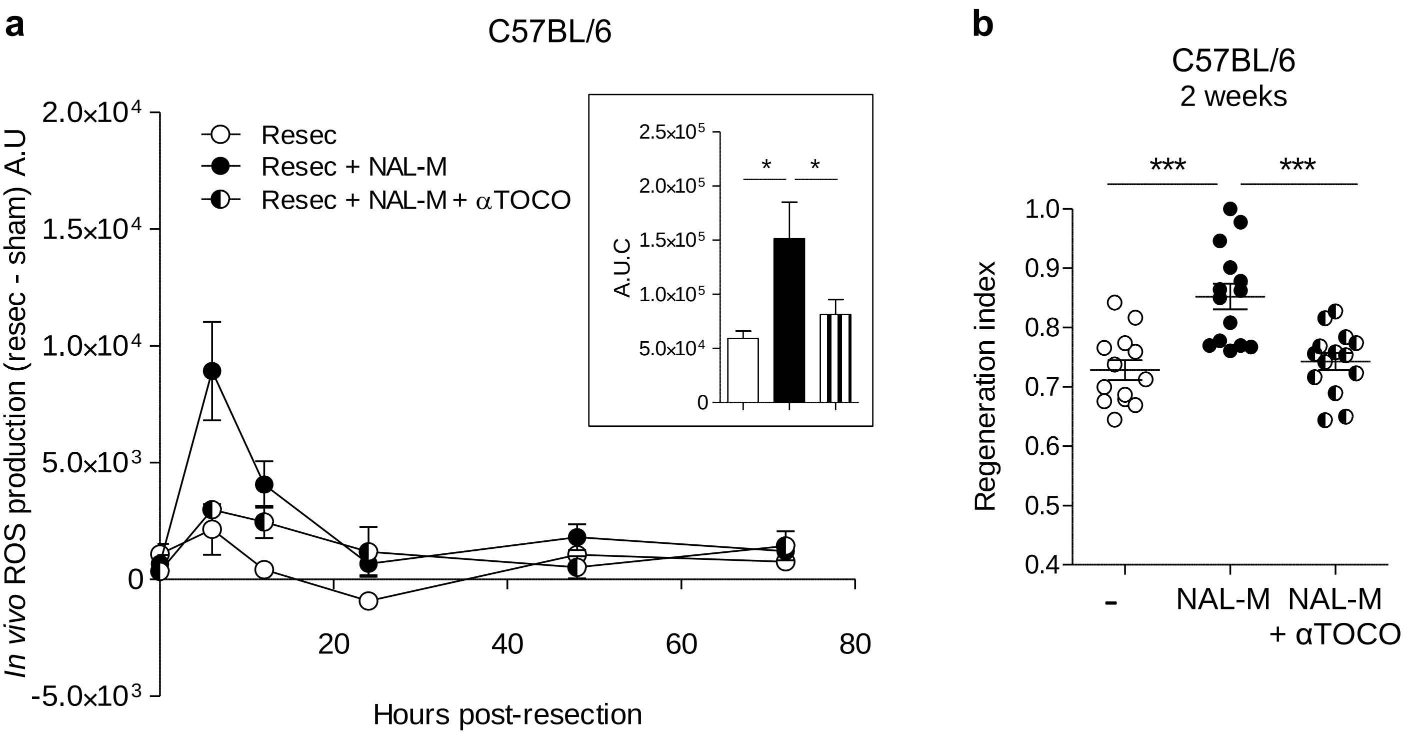

Figure S3: Opioid antagonist increases ROS production specifically in CD45 positive cells.

In vitro quantification of ROS production in $CD45^-$ (a) or $CD45^+$ (b) cells sorted from IFP stromal vascular fraction. Cells were activated with PMA (phorbol-12-myristate-13-acetate) and treated (black bar) or not (striped bar) with NAL-M. One representative experiment of three independent experiments is presented here, n=3 animals per group. Data are represented as mean ± SEM (n.s: not significant, * p<0.05). NAL-M: naloxone methiodide.

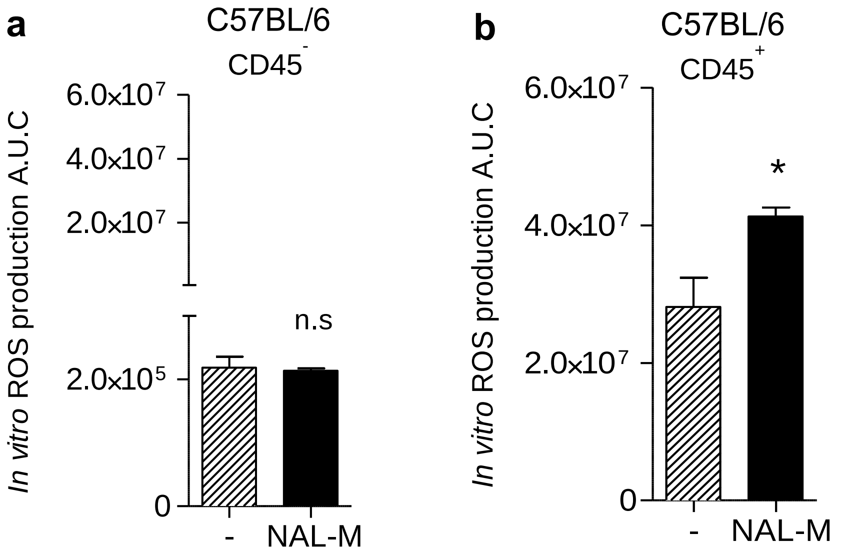

Supplement: Supplementary file 1 — Supplementary Information [file 41598_2018_29594_MOESM1_ESM.pdf]
